# Supplementary material for: What’s in a Name? Patterns, Trends, and Suggestions for Defining Non-Perennial Rivers and Streams
Source: Water (Basel). Author manuscript; Available in PMC 2021 Jul 13. (PMC7707420)
Supplement: Supplement1 [file NIHMS1639359-supplement-Supplement1.zip › suppl_final/suppl/water-835375-for suppl.docx]

*Review*

**What’s in a Name? Patterns, Trends, and Suggestions for Defining Non-Perennial Rivers and Streams**

Michelle H. Busch ^1,^*, Katie H. Costigan ^2^, Ken M. Fritz ^3^, Thibault Datry ^4^,
Corey A. Krabbenhoft ^5^, John C. Hammond ^6,†^, Margaret Zimmer ^7^, Julian D. Olden ^8^,
Ryan M. Burrows ^9^, Walter K. Dodds ^10^, Kate S. Boersma ^11^, Margaret Shanafield ^12^,
Stephanie K. Kampf ^13^, Meryl C. Mims ^14^, Michael T. Bogan ^15^, Adam S. Ward ^16^,
Mariana Perez Rocha ^17^, Sarah Godsey ^18^, George H. Allen ^19^, Joanna R. Blaszczak ^20^,
C. Nathan Jones ^21^ and Daniel C. Allen ^1^

Supplemental Materials

S1. Example Search

Each epithet (Table 1) had an individual Clarivate Web of Science search done to collect the abstracts and papers used in the analyses. To limit search results to non-perennial river systems we limited the paired water body term to 41 based on conversation between authors. We also limited our search to 37 WoS categories. As an example, the search for epithet “arid” was:

TS = (*arid* NEAR/0 (river* OR stream* OR wadi* OR flow* OR "dry bed*" OR corridor* OR riverbed* OR branch* OR run* OR fork* OR brook* OR kill* OR bayou* OR swamp* OR wash* OR cañada* OR arroyo* OR rio* OR crik* OR creek* OR allt* OR water* OR burn* OR beck* OR afron* OR canal* OR prong* OR slough* OR lick* OR drain* OR coulee* OR outlet* OR ditch* OR waterbod* OR channel* OR rill* OR gull* OR tributar* OR hydrograph* OR headwater* OR watershed*) AND WC = (agricultural engineering OR agriculture multidisciplinary OR agronomy OR biodiversity conservation OR biology OR chemistry applied OR chemistry physical OR computer science interdisciplinary applications OR ecology OR engineering civil OR engineering environmental OR entomology OR environmental sciences OR environmental studies OR evolutionary biology OR fisheries OR forestry OR geography physical OR geology OR geosciences multidisciplinary OR green sustainable technology horticulture OR limnology OR marine freshwater biology OR materials science multidisciplinary OR meteorology atmospheric sciences OR microbiology OR multidisciplinary sciences OR oceanography OR physics applied OR physics multidisciplinary OR plant sciences OR public environmental occupation health OR remote sensing OR soil science OR water resources OR zoology).


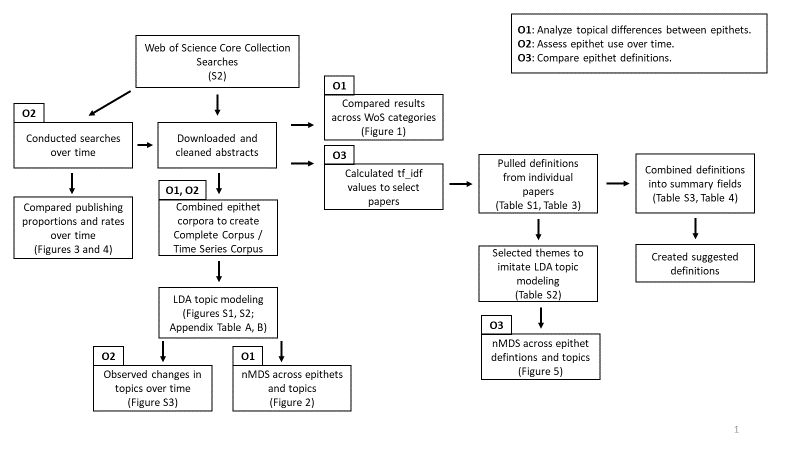


**Figure S1.** Methods Flow Chart.

A methods flow chart was created to provide additional clarity. All figures, tables, supplementary materials, and appendices are included in the flow chart to provide context. O1, O2, and O3 refer to the Objective number as listed in the manuscript. Full objectives are included within the flow chart to provide a complete overview of our analyses.


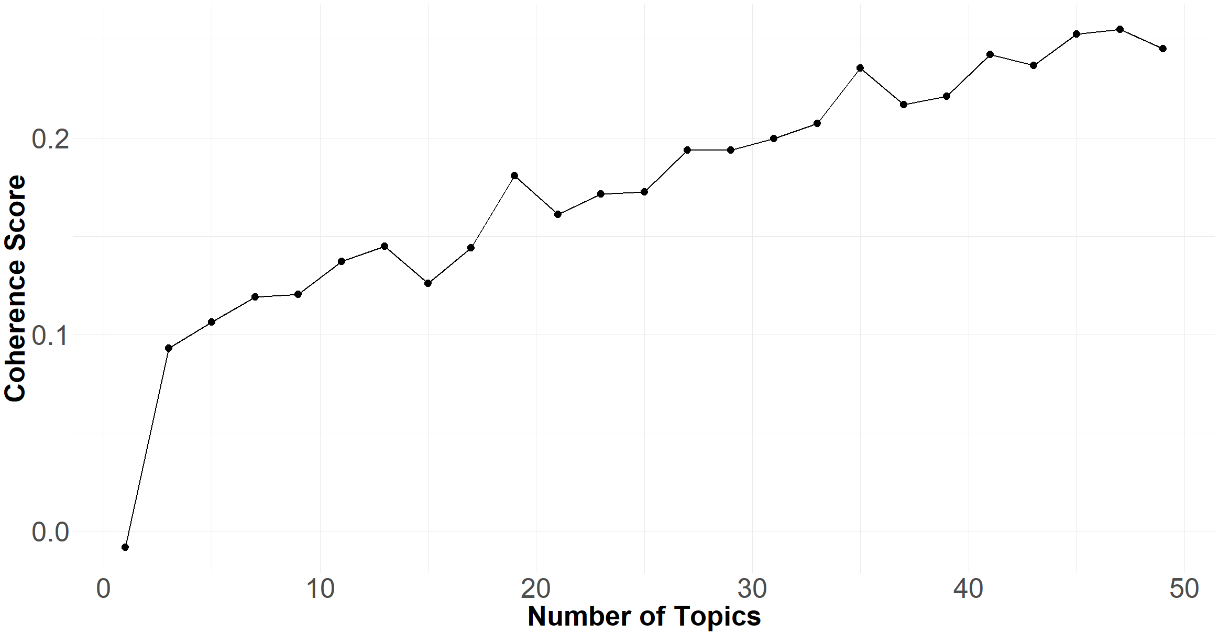


**Figure S2.** Coherence scores over number of topics for Complete Corpus.

A matrix was created to evaluate coherence scores from 1 to 50 topics by multiples of two before running LDA topic models. We selected six topics to explore further. While the coherence values continued to rise after 6 topics, the lack of a clear plateau in the graph indicated that coherence values could continue to rise with an increase in the number of topics. When exploring a higher number of topics, we found multiple cases of overlap where several of the same words were used for an increasing number of topics (for example, ten words that made up topic number 3 were also found to make up topic number 8). We chose 6 topics as the coherence value crossed the 0.1 threshold as well as a need to have a manageable number of topics to work with.


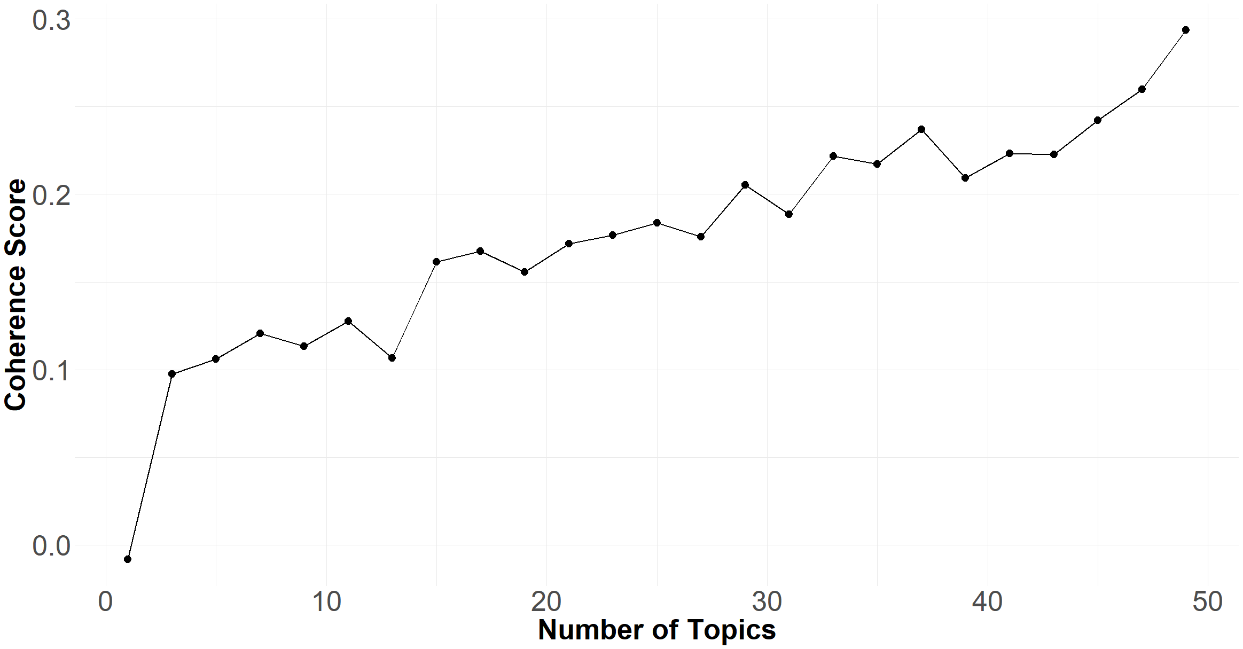


**Figure S3.** Coherence scores over number of topics for Time Series Corpus.

A matrix was created to evaluate coherence scores from 1 to 50 topics prior to running LDA topic modeling. We selected nine topics to explore further; once again based on a lack of a plateau, overlap of topics, and on the coherence plot while wanting a reasonable number of topics.


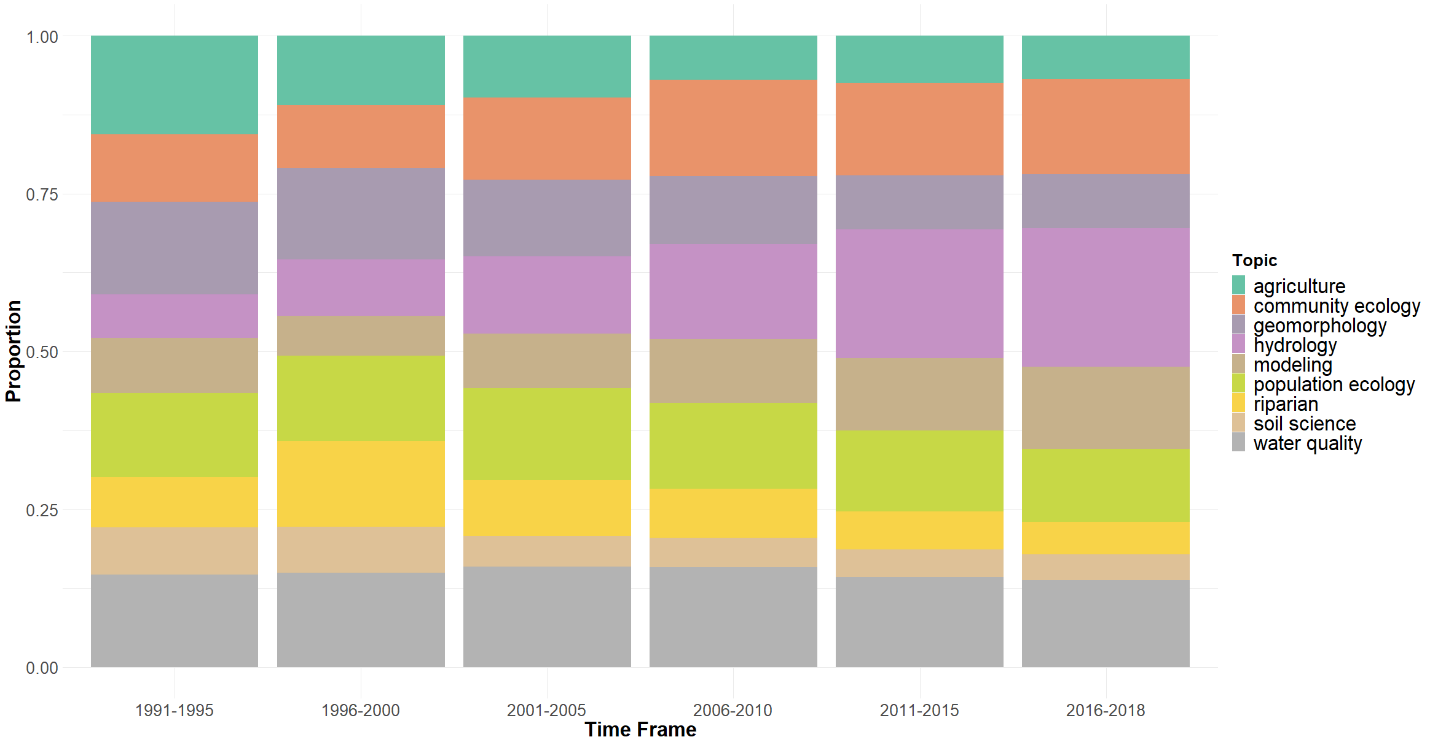


**Figure S4.** Time Series topics over time.

The proportion of each topic from the Time Series Corpus LDA topic modeling between 1991 and 2018. Colors represent different topics, named after reviewing the top 20 terms associated with each topic (Appendix B).

**Table 1.** Papers used in definition analysis.

The final list of papers used in our definition analysis grouped by epithet. A maximum of 50 papers were included in our original definition mining search. These original papers were limited to only using waterbody terms “river” and “stream” to exclude papers not about non-perennial rivers. Due to the lack of papers that held definitions in our original search, we included an addition randomly selected 25 papers that were not limited by waterbody terms. This gave us a total of 672 papers for definition mining. Authors were limited to include the first four in the spreadsheet. (Please see Excel file “TableS1-Definition Papers.csv”)

**Table S2.** Definition analysis themes.

| **Broad Theme** | **Specific Theme** |
| --- | --- |
| Source | Precipitation / Runoff |
| Source | Groundwater |
|  | Seasonality / Predictability |
|  | Variability / Unpredictability |
|  | Linked to Specific Timeframe |
|  | Linked to Specific Landscape |
|  | Related to Extremes (Floods / Droughts) |
| Phases of Drying | Low Flow |
| Phases of Drying | No Flow |
| Phases of Drying | Isolated Pools |
| Phases of Drying | No Surface Water |
| Phases of Drying | Not Specific |

As definition corpora were too small to run LDA topic modeling, we selected the following themes that are common across non-perennial literature. Some themes were more related to each other than others, which is noted by the Broad Theme column.

**Table S3.** Web of Science categories by research field.

| **WoS Category** | **Summary Field** |
| --- | --- |
| Ecology | Ecology |
| Water Resources | Hydrology |
| Civil Engineering | Hydrology |
| Geosciences | Hydrology |
| Geography | Hydrology |
| Environmental Studies | Eco-Hydrology |
| Limnology | Eco-Hydrology |
| Biodiversity and Conservation | Eco-Hydrology |
| Sustainability | Eco-Hydrology |

WoS assigned categories for papers and the resulting summary fields they were placed into. Once mined definitions were placed into summary fields, definitions were reviewed to create summary definitions.
